# The Role of *CYP2D6* Polymorphisms in Determining Response to Tamoxifen in Metastatic Breast Cancer Patients: Review and Egyptian Experience

**DOI:** 10.31557/APJCP.2020.21.12.3619

**Published:** 2020-12

**Authors:** Ibrahim Malash, Osman Mansour, Sabry Shaarawy, Mona S Abdellateif, Anan Omar, Rabab Gaafar, Abdel-Rahman N Zekri, Ola S Ahmed, Abeer Bahnassy

**Affiliations:** 1 *Medical Oncology department, National Cancer Institute (NCI), Cairo University, Egypt. *; 2 *Medical Biochemistry and Molecular Biology, Egypt. *; 3 *Molecular Virology and Immunology Unit, Cancer Biology Department, NCI, Cairo University, Egypt. *; 4 *Tissue Culture and Cytogenetics Unit, Department of Pathology, National Cancer Institute, Cairo University, Egypt. *

**Keywords:** MBC, CYP2D6, polymorphisms, Tamoxifen, response to treatment

## Abstract

**Background:**

Metastatic breast cancer (MBC) represents a major health problem in Egypt and worldwide. Prognostic and predictive factors for patients with MBC are highly required for better management and improved survival. The aim of this study was to assess the prognostic and predictive value(s) of *CYP2D6 *polymorphisms in Tamoxifen responders and non-responders.

**Methods:**

A cohort of 157 hormone receptor positive, locally recurrent inoperable and/or metastatic (MBC) Egyptian female patients was assessed for *CYP2D6 *polymorphisms. Data were correlated to relevant clinic-pathological features of the patients, response to tamoxifen, and survival rates.

**Results:**

*CYP2D6* polymorphisms were detected in 44/157 cases (28%), 30 of them (68.2%) were refractory and 14 (31.8%) were responders (P=0.027). The CYP2D6 *3,*4 variants were significantly prevalent in the refractory group 26/30 (86.6%), while the *10/*10 and *10/*3 variants were more common in the responders 12/14 (85.71%, P=0.027). CYP2D6 polymorphism associated significantly with Her-2 amplification (P=0.001) as well as reduced overall survival rates in both refractory and responder patients (P< 0.001).

**Conclusion:**

*CYP2D6* polymorphisms can significantly predict response to Tamoxifen treatment, and also associates with poor overall survival rates in MBC patients.

## Introduction

Breast cancer (BC) is the second-leading cause of cancer-related death among women worldwide (Siegel et al., 2016). It is a heterogeneous disease with a varied response to treatment and survival rates. In Egypt, BC represents ~38.2% of all female malignancies with more than 60% of BC patients expressing estrogen receptors (ER) and/or progesterone receptors (Ibrahim et al., 2014). These patients are candidates for hormonal therapy that blocks estrogen signaling in BC cells (Moy and Goss, 2006). Tamoxifen is a selective estrogen receptor modulator (SERM) which is usually used for early as well as advanced ER/PR BC patients. Moreover, Tamoxifen improves both relapse-free and overall survival rates significantly in those patients (Fisher et al., 1998). Nowadays, more than 400,000 women worldwide are treated with Tamoxifen therapy, and other millions have benefited from palliation and extended disease-free survival (Jordan, 2003).

During the last few years, several mechanisms have been proposed to explain the acquired resistance to Tamoxifen including activation of specific molecular pathways; such as EGFR and/or HER-2 signaling, RAS/RAF/MEK/MAPK signaling, PI3`K/AKT, mTOR or PTEN signaling pathways (Clarke et al., 2003). Recent studies which assessed these pathways provided evidence that there are several predictive biomarkers for therapeutic response to hormonal therapies for MBC patients. Therefore, gene expression analysis and molecular profiling techniques are expected to define gene signatures that could predict clinical responses to treatment, and identify new therapeutic options (Musgrove and Sutherland, 2009; Riggins et al., 2007). 

The CYP2D6 is an important member of the enzyme cytochrome P450 family, which is responsible for drug metabolism. It has been proposed that genetic polymorphism(s) in CYP2D6 (the Tamoxifen-metabolizing genes) affect the plasma concentration of Tamoxifen metabolites, because CYP2D6 poor metabolizers contain two null alleles, which produce the lowest plasma active metabolite concentrations. On the other hand, the rapid metabolizer patients have duplicate or multi-duplicate gene copies of the same allele. This will induce increased enzyme activity and consequently produce the highest concentrations of active metabolite in the plasma (Brauch and Schwab, 2014). In addition, patients with certain genotypes of cytochrome p450 2D6 (CYP2D6) are more likely to have a higher risk of disease relapse, suggesting that detection of CYP2D6 polymorphism(s) could identify patients who will get the maximum benefit from Tamoxifen therapy (Johnston, 2006). 

According to the literature, *CYP2D6 *Polymorphism varied widely between different races and populations (Johnston, 2006; Qian et al., 2013; He et al., 2016). Therefore, the aim of the current study was 1) To assess the possible prognostic and predictive value(s) of *CYP2D6 *polymorphisms in MBC patients treated with Tamoxifen through determining the impact of these polymorphisms on patients’ response to Tamoxifen and survival rates. 2) To compare between our data regarding the frequency and patterns of *CYP2D6* polymorphisms with other reported studies on different populations. This could help to identify a potential target for therapies that could be used to alleviate Tamoxifen resistance and improve the clinical outcomes (in the form of recurrence and survival) in Egyptian MBC patients. 

## Materials and Methods

Patients: this is a prospective cohort study which included 157 MBC patients who were diagnosed and treated at the National Cancer Institute, Cairo University during the period from January 2014 to December 2016. All patients had metastatic or locally recurrent inoperable disease and none of them received prior hormonal therapy (Tamoxifen). Patients with rapidly progressive and/or large volume visceral metastases, central nervous system (CNS) metastases, or inflammatory breast cancer were excluded from the study. Similarly, patients with contra-indication to Tamoxifen and those with prior deep venous thrombosis and/or anti-coagulant medication within 2 weeks of registration were excluded. Concomitant pain medications were freely allowed, as well as local palliative radiotherapy for painful or high risk bone metastases. Bisphosphonates were also freely allowed in case of bone involvement whenever indicated. 

All patients were afforded baseline assessment including history and physical examination, evaluation of all potential tumor sites (by chest X-ray, bone scan, liver imaging and serum tumor markers) routine biochemistry and hematology tests. Patients were also assessed for any adverse events and tumor response after 4, 8, 16 and 24 weeks of treatment. All involved disease sites were re-evaluated and target lesions were measured at each tumor evaluation (except for the bone scan which was repeated at week 24 only). At week 4, only local disease was assessed and tumor markers were repeated if initially elevated. Determination of the performance status and physical examination were also done. Patients were then categorized into; those who achieved and maintained good clinical response to Tamoxifen for more than 6 months (Tamoxifen responders), and those whose cancer progressed during the first 6 months of treatment (Tamoxifen resistant). 

*CYP2D6 polymorphism*

DNA was extracted from formalin-fixed- paraffin-embedded breast tissue samples (FFPETs) and peripheral blood lymphocytes of BC patients as well as from the normal control subjects (blood donors) using the DNA extraction kit (QIAGEN) according to manufacturer’s instructions. polymerase chain reaction (PCR) and restriction enzyme cut BstNI were performed for the identification of CYP2D6 *3, *4, *41 polymorphism (Raimundo et al., 2004; Zanger et al., 2004; Okishiro et al., 2009). The TaqMan single nucleotide polymorphism (SNP) Genotyping Assays (Applied Biosystems, Foster City, California) was performed for identification of the CYP2D6 *10 allele. Accordingly, cases were classified into: CYP2D6 wt/wt, CYP2D6 *10/wt or CYP2D6 *10/*10. 

*Statistical Analysis*

Data were analyzed using SPSS version 21(IBM SPSS, Armonk, NY, USA). Qualitative data were described as frequencies and percentages. The relation between qualitative data was determined using the Chi-square test or the non- parametric Fisher’s exact test as appropriate. Comparison between survival distributions of different groups was performed by the log rank test. Probability (P value) equal or less than 0.05 was considered significant.

## Results

Clinico-pathological features of the patients: The mean age of the patients assessed was 51.63±10.5 years and the median was 52 years. Eighty-one patients (51.6%) were >50 years and 76(48.4%) were <50 years old. The minimum age of the assessed patients was 29 years, the maximum was 79 years, and nine patients were less than 35 years. Thirty-one of all patients (19.7%) gave history of previous hormonal contraceptive pills, 73 patients (46.5%) were premenopausal and 84 (53.5%) were postmenopausal. All patients presented with ECOG performance status ≤2 and they were almost equally distributed between the 2 groups (responders and refractory, Table 1).

Response to Tamoxifen treatment: Among the 157 patients assessed, 78 patients (49.7%) responded to Tamoxifen treatment, and 79 (50.3%) were refractory. There was a significant association between patient’s response to Tamoxifen and the site of metastasis. patients with only bone metastasis demonstrated noticeable good and maintained response to Tamoxifen compared to those patients who have visceral metastases, since out of the 46 patients who had bone metastasis only, 15(32.6%) were refractory compared to 31(67.4%) who were tamoxifen responders. On the other hand, out of the 111 patients who had visceral metastasis, 64 (57.7%) were refractory and 47 (42.3%) were responders; P=0.005). None of the other clinico-pathological features assessed in the current study including the age, menstrual status, use of contraceptive pills, tumor size, histological grade or *Her-2* gene status correlated significantly with response to Tamoxifen (Table 2).

**Figure 1:**
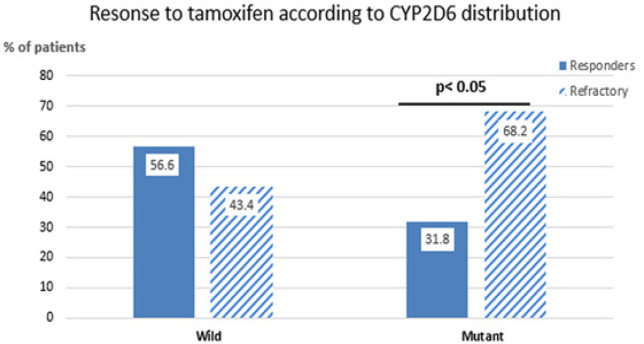
CYP2D6 Polymorphism in Responders and Refractory Group

*CYP2D6* polymorphism(s) analysis: The wild type CYP2D6 (WT) was present in 113 (71.97%) of the patients [49/79 (62.02%) in the refractory group and 64/78 (82.1%) in the responders]. On the other hand, *CYP2D6 *polymorphisms were detected in 44 cases (28%), 30 of them (68.2%) were refractory and 14 (31.8%) were responders (P=0.027; Figure 1). The most common *CYP2D6 *polymorphic variants detected in the refractory group were CYP2D6 *3 and *4 polymorphisms, which were present in 26/30 (86.66%) of the patients, whereas the remaining 4/30 (13.3%) patients showed CYP2D6 *10/*10 in one patient (3.33%) and CYP2D6 *wt/*10 in three patients (10%). In the group of responders, CYP2D6 *10/*10 and CYP2D6 *10/*3 were present in 12/14 (85.71%) patients, whereas the remaining two patients (14.3%) had *41/*41 and *wt/*41 polymorphic variants (Table 3). 

Correlation between *CYP2D6* polymorphisms and the relevant characteristics of the patients: There was significant correlation between *CYP2D6* polymorphisms and PR expression, since out of the 14 tamoxifen responder patients with *CYP2D6* polymorphisms, 11 patients (78.6%) were PR +ve, and 3 (21.4%) were PR –ve (P=0.012), compared to the tamoxifen refractory patients with* CYP2D6* polymorphisms, 11 patients (36.7%) were PR +ve, and 19 (63.3%) were PR –ve (P=0.08). *CYP2D6* polymorphisms was also more common in responder patients whose age more than 50 years old, since out of the 14 patients with mutant CYP2D6, 10 patients (71.4%) were ≥50 years, and only 4 (28.6%) were <50 years old (P=0.04). Similarly, HER2-neu protein expression associated significantly with *CYP2D6* polymorphisms, since all responding patients who had *CYP2D6* polymorphisms, showed HER2-neu overexpression [14/14 (100%), P=0.001]. whereas, out of the 64 responders with wild type CYP2D6, 17 (26.6%) showed HER2-neu overexpression (P=0.001). The same was also true in the refractory group, since HER2-neu overexpression was present in 18/30 (60%) of patients with *CYP2D6* polymorphism, compared to 9/49 (18.4%) only in patients with wild type CYP2D6 (P=0.001, Table 5). 

*Correlation between CYP2D6 polymorphisms and the survival rate of patients*

**Figure 2:**
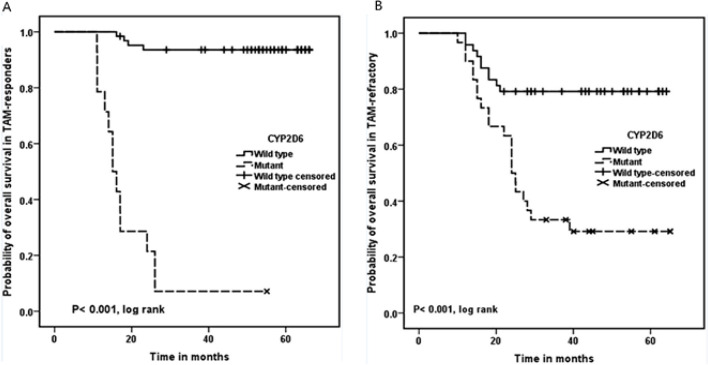
Correlation between CYP2D6 Polymorphisms and the Survival of; A) Tamoxifen responders , B) Tamoxifen refractory patients

There was a statistically significant association between CYP2D6 polymorphism(s) and reduced overall survival rate of the patients in both responder and refractory groups (P< 0.001 for both; Figure 2A, B).

**Table 1:** Relevant Clinico-Pathological Features of the Assessed Breast Cancer Patients

Characteristics	N=157 (%)
Age	
Mean: 51.63±10.5	
<50	76 (48.4)
≥50	81 (51.6)
Tumor size	
≤ 5cm	131 (83.4)
> 5 cm	26 (16.6)
TNM Staging-T	
T1	13 (8.3)
T2	104 (66.2)
T3	40 (25.5)
Lymph Nodes	
N0	30 (19.1)
N1	51 (32.5)
N2	51 (32.5)
N3	25 (15.9)
Grade	
1	11(7)
2	112 (71.3)
3	34 (21.65)
ER	
Negative	49 (31.2)
Positive	108 (68.8)
PR	
Negative	79 (50.3)
Positive	78 (49.7)
HER2	
Negative	99 (63.1)
Positive	58 (36.9)
CYP2D6 polymorphisms	
Wild	113 (71.97)
Mutant	44 (28)

**Table 2:** Patients’ Characteristics and Their Relation to Tamoxifen Response

Feature	Refractory (79)	Responders (78)	P-value
Age (years)			
>50 (81)	41	40	1
≤50 (76)	38	38	
Menstrual Status			
Pre (73)	37	36	1
Post (84)	42	42	
Histological grade			
II (114)	57	57	0.69
III (43)	22	21	
Contraception			
Yes (31)	14	17	0.55
No (126)	65	61	
Her-2 status			
+ve (58)	27	31	0.51
-ve (99)	52	47	
ER			
+ve (108)	56	52	0.61
-ve (49)	23	26	
PR			
+ve (78)	40	38	1
-ve (79)	39	40	
Disease site			
Bone only (46)	15	31	0.005
Visceral (111)	64	47	
CYP2D6 status			
Mutant (44)	30	14	0.027
Wild (113)	49	64	

**Table 3:** The Frequency of CYP2D6 Polymorphisms in Responders and Refractory Patients

CYP2D6 polymorphisms	Responding group (14)	Refractory group (30)
CYP2D6 *3/ *4	0%	26 (86.66%)
CYP2D6 *wt/*10	0%	3 (10%)
CYP2D6 *10/*10 & CYP2D6 *10/*3	12 (85.71%)	1 (3.33%)
CYP2D6*41/*41 & CYP2D6*wt/*41	2 (14.3%)	0%

**Table 4:** Correlation between CYP2D6 Polymorphism and Patients’ Characteristics in Responders and Refractory Groups

Characteristics	Responders		P value	Refractory		P value
	Wild (64)	Mutant (14)		Wild (49)	Mutant (30)	
Age						
Mean: 53.26±10.41						
<50 (38)	34 (53.1%)	4 (28.6%)	0.04	38(77.6%)	12 (40%)	0.48
≥50 (40)	30 (46.9%)	10 (71.4%)		11 (22.4%)	18 (60%)	
Tumor size						
≤ 5cm (61)	50(78.1%)	10 (71.4)	0.57	46 (93.8)	25 (83.3)	0.27
> 5 cm (18)	14 (21.9%)	4 (28.6)		3 (6.2)	5 (16.7)	
TNM staging						
T						
T1 (5)	4 (6.3%)	1 (7.1)		5 (10.2)	3 (10)	0.73
T2 (49)	40 (62.5%)	9 (64.3)	0.98	33 (67.4)	22 (73.3)	
T3 (24)	20 (31.2%)	4 (28.6)		11 (22.4)	5 (16.7)	
T4 (0)	0 (0.0)	0 (0.0)		0 (0)	0 (0)	
N						
N0 (8)	8 (12.5)	0 (0.0)		14 (28.6)	8 (27.7)	0.71
N1 (20)	17 (26.6)	3 (21.4)	0.5	20 (40.8)	11 (36.7)	
N2 (32)	25 (39.0)	7 (50.0)		12 (24.5)	7 (23.3)	
N3 (18)	14 (21.9)	4 (28.6)		3 (6.1)	4 (13.3)	
Grade						
1 (4)	4 (6.2)	0 (0.0)	0.57	5 (10.2)	2 (6.7)	0.52
2 (46)	38 (59.4)	8 (57.1)		39 (79.6)	27 (86.6)	
3 (28)	22 (34.4)	6 (42.9)		4 (8.2)	2 (6.7)	
ER						
-ve (26)	23 (35.9)	3 (21.4)	0.27	14 (28.6)	9 (30)	0.96
+ve (52)	41 (64.1)	11 (78.6)		35 (71.4)	21 (70)	
PR						
-ve (40)	37 (57.8)	3 (21.4)	0.012	20 (40.8)	19 (63.3)	0.08
+ve (38)	27 (42.2)	11 (78.6)		29 (59.2)	11 (36.7)	
HER2						
-ve (47)	47 (73.4)	0	0.001	40 (81.6)	12 (40)	0.001
+ve (31)	17 (26.6)	14 (100)		9 (18.4)	18 (60)	

## Discussion

Tamoxifen is an effective drug for the treatment of hormonal positive breast cancer patients. However, about 30%–50% of those patients may develop relapse. Previous studies provided evidence that allelic variations in cytochrome P450-2D6 enzyme highly affect response to tamoxifen by modulating the metabolism of tamoxifen into its pharmacologically active metabolite endoxifen (Wickramage et al., 2017). Although *CYP2D6 *polymorphisms has been investigated in many studies in the western countries, the results of its role on tamoxifen concentrations as well as its impact on the clinical outcomes of the treated patients are conflicting and still not clear (Markopoulos et al., 2014). Therefore, we sought to assessed the possible prognostic and predictive role(s) of *CYP2D6* polymorphisms in metastatic and/or locally recurrent inoperable breast cancer patients from Egypt who were treated with Tamoxifen. These could help us to identify patients who will not respond to tamoxifen treatment and accordingly they should shift to other therapeutic modalities that help to improve clinical outcomes in those non-responding patients.

It has been reported that CYP2D6 status varies dramatically among different populations and ethnic groups. In our studied Egyptian patients, we found that *CYP2D6* polymorphisms were present in 28% of the assessed cases. The most commonly detected CYP2D6 polymorphic variant was CYP2D6 *3 /*4. However, He et al., (2016), reported that CYP2D6*17 variant is mainly found in African populations, and the CYP2D6*41 was found in African and Caucasian populations. On the other hand, other studies from Asia demonstrated that CYP2D6*10 was the most prevalent polymorphic variant in Asian populations, with a frequency of 30–50% in Chinese populations (Qian et al., 2013; Helland et al., 2017; Lan et al., 2018). Finally, CYP2D6*4 was detected mainly in Caucasians with a frequency of 20–25% (Bradford, 2002).

Taken together, the current study demonstrates that *CYP2D6* polymorphisms associated significantly with poor response to treatment and decreased overall survival rates of the patients. Our data in this context are in agreement with many published studies in literature which reported that Reduced CYP2D6 activity is usually associated with poor treatment outcomes, increased risk of recurrence and shortened recurrence as well as with overall free survival rates in breast cancer patients treated with Tamoxifen (Thota et al., 2018, Kiyotani et al., 2010). However, these data are contradictory to the previously published data of Hertz (2017), who performed a retrospective analysis of two community-based studies to assess for the associations between the low-activity CYP2D6 genotype and disease outcome in 500 breast cancer patients treated with adjuvant tamoxifen monotherapy, and 500 patients who did not receive any systemic adjuvant therapy. According to their data, they concluded that there was no significant association between the CYP2D6 genotype and the outcomes of patients on tamoxifen treatment. Another interesting study by Brooks et al, performed on 1,514 patients with contralateral breast cancer compared to 2203 patients with unilateral breast cancer from USA. They concluded that CYP2D6 phenotype may have a role in the risk of developing contralateral breast cancer in patients treated with tamoxifen (Brooks et al., 2018).

Additionally, our data revealed that the most frequent polymorphic variants in the refractory group were CYP2D6 *3, *4 which represent the commonest type in the poor metabolizers. Moreover, those patients with the CYP2D6 polymorphic variants have more aggressive tumor, lower overall survival, and no response to tamoxifen treatment. These data are consistent with those of Goetz et al., (2005), who found that patients with the CYP2D6*4 genotype had poor prognosis, and higher risk of disease relapse. Similarly, Schroth et al., (2007) and Thota et al., (2018), supported those studies by reporting that CYP2D6*4 genotype can identify patients who will have little benefit from adjuvant tamoxifen therapy. Bradford (2002), also confirmed these data and concluded that CYP2D6*4 is responsible for 70–90% of all poor metabolizers in Caucasian patients.

As for responders, the most common CYP2D6 polymorphic variants were CYP2D6 *10/*10 and CYP2D6 *10/*3, while CYP2D6*41/*41 and CYP2D6*wt/*41 represented only 14.3%. these data are in agreement with Okishiro et al., (2009), who mentioned that CYP2D6 *10 didn’t associate with poor outcomes of a cohort of Japanese BC patients. However, this finding was not in accordance with other recent studies reported that CYP2D6*10 genotype is more common in the intermediate and poor metabolizer BC patients from south Indian (Kiyotani et al., 2010), and china (Xu et al., 2008; Zeng et al., 2017; Wei et al., 2020), with increased recurrence and reduced survival rates. A meta-analysis done by Bai et al, on a cohort studies published before March 2018 in English and Chinese databases. They demonstrated that patients with TT genotype of CYP2D6*10 had lower concentrations of 4-OH-TAM than those with the CC genotype (Bai et al., 2019).

Taken together, these data emphasize the importance of tailoring Tamoxifen treatment according to CYP2D6 genotypes, which might differ among ethnic groups and geographic areas. Accordingly, further studies on Egyptian patients are needed in this area to establish such an association with high statistical power, because the Egyptian population contains different ethnic groups (e.g. upper and lower Egypt, etc). The importance of this point has been clarified by the data of Wei et al., (2020), from the perspective of the Chinese healthcare system, who concluded that CYP2D6 testing was cost effective for postmenopausal women with ER-positive early breast cancer.

In the current study, we were not able to find significant correlation between Tamoxifen response and relevant clinic-pathological features of the assessed patients such as the age of patients, histological grade or stage, menopausal status, LN metastasis, etc. However, we found that patients with bone only disease (regardless of other studied factors), showed a good and maintained response to Tamoxifen compared to those with visceral involvement. The difference between both groups was statistically significant. Accordingly, Tamoxifen had to be given to patients with bone only disease whenever possible.

Our results showed that *CYP2D6* polymorphisms associates significantly with Her-2 –amplification, as it was present in 60% of the refractory group, and in all responding patients with mutant *CYP2D6*. Even though it was not in agreement with Okishiro (2009), who reported no significant association detected between *CYP2D6 *polymorphisms and *Her-2 *–amplification in the Japanese BC patients. 

At the end, we concluded that *CYP2D6* polymorphism(s) could be used to predict response to Tamoxifen, and reduced overall survival in metastatic BC patients. Moreover, *CYP2D6* polymorphisms varies between different populations from different area in the world. therefore, more studies are required to identify the exact variant of *CYP2D6* polymorphisms and also the relevant enzymes in the studied breast cancer patients. 
